# Anti-inflammatory effects of tetradecylthioacetic acid (TTA) in macrophage-like cells from Atlantic salmon (Salmo salar L.)

**DOI:** 10.1186/1471-2172-12-41

**Published:** 2011-07-20

**Authors:** Fabian Grammes, Harald Takle

**Affiliations:** 1 Institute of Animal and Aquaculture Sciences, Norwegian University of Life Sciences, P.O. Box 5003, N-1432 As-UMB, Norway; 2 NOFIMA, P.O. Box 5010, N-1432 s, Norway; 3 AVS Chile SA, Casilla 300, Puerto Varas, Chile

## Abstract

**Background:**

Commercial Atlantic salmon is fed diets with high fat levels to promote fast and cost-effective growth. To avoid negative impact of obesity, food additives that stimulate fat metabolism and immune function are of high interest. TTA, tetradecylthioacetic acid, is a synthetic fatty acid that stimulates mitochondrial *β*-oxidation most likely by activation of peroxysome proliferator-activated receptors (PPARs). PPARs are important transcription factors regulating multiple functions including fat metabolism and immune responses. Atlantic salmon experiments have shown that TTA supplemented diets significantly reduce mortality during natural outbreaks of viral diseases, suggesting a modulatory role of the immune system.

**Results:**

To gain new insights into TTA effects on the Atlantic salmon immune system, a factorial, high-throughput microarray experiment was conducted using a 44K oligo nucleotide salmon microarray SIQ2.0 and the Atlantic salmon macrophage-like cell line ASK. The experiment was used to determine the transcriptional effects of TTA, the effects of TTA in poly(I:C) elicited cells and the effects of pretreating the cells with TTA. The expression patterns revealed that a large proportion of genes regulated by TTA were related to lipid metabolism and increased mitochondrial *β*-oxidation. In addition we found that for a subset of genes TTA antagonized the transcriptional effects of poly(I:C). This, together with the results from qRT-PCR showing an increased transcription of anti-inflammatory *IL10 *by TTA, indicates anti-inflammatory effects.

**Conclusions:**

We demonstrate that TTA has significant effects on macrophage-like salmon cells that are challenged by the artificial dsRNA poly(I:C). The immune stimulatory effect of TTA in macrophages involves increased lipid metabolism and suppressed inflammatory status. Thus, suggesting that TTA directs the macrophage-like cells towards alternative, anti-inflammatory, activation. This has positive implications for TTA as a feed additive.

## Background

In commercial Atlantic salmon (*Salmo salar *L.) aquaculture, diets usually contain high levels of fat as it provides an inexpensive source of energy and promotes rapid growth. However, high levels of dietary fat cause excess fat to be deposited in the tissues, most notably in muscle, liver and as visceral fat [1]. Thereby the diet may affect the general health of the Atlantic salmon and also the quality of the filet as the final product [2]. In order to reduce the negative side effects of high fat diets, several studies have experimented with food additives promoting fat catabolism. Tetradecylthioacetic acid (TTA) is one of these additives, considered to be highly interesting.

TTA is a synthetic fatty acid with a sulfur substitution at the 3rd position in the carbon chain. The chemical properties of TTA resemble those of a normal fatty acid of similar length. What makes TTA interesting is that the sulfur atom makes it resistant towards further catabolism via *β*-oxidation and that TTA serves as activator for all peroxysome proliferator-activated receptor (PPAR) subtypes [3,4]. PPARs are important transcription factors regulating multiple functions, most notably fat metabolism and immune responses (reviewed in [5]). TTA has been reported to induce pleiotropic effects in mammals. These effects include increased mitochondrial *β*-oxidation in muscle and liver, decreased plasma lipid levels as well as antioxidant and anti-inflammatory effects (reviewed in [6,7]). It appears that most of the metabolic effects of TTA are mediated through activation of PPARs.

Studies addressing metabolic effects of TTA in fish are scarce. Nevertheless, the studies that have been conducted with members of the salmonidae family indicate similar metabolic effects of TTA in fish by showing increased lipid metabolism in liver [8,9] and muscle [10]. In contrast to mammals, high levels of dietary TTA have been reported to induce mortality in Atlantic salmon [9,11] but not in Rainbow trout (*Oncorhynchus mykiss *Walbaum) [8]. It has been hypothesized that the mortality may be related to accumulation of TTA-metabolites in the kidney at low temperatures [12].

To date only one study investigated immune modulatory functions of TTA in Atlantic salmon, showing that TTA reduces the production of leukotriene B4 (LTB_4_) and prostaglandin E_2 _(PGE_2_) in headkidney macrophages isolated from TTA fed fish [12]. It should be noted that the reduction was found only to be significant for cells cultured at 5°C, but not in those maintained at 12°C [12]. Intriguingly, two studies, both reporting significant reduced mortality in Atlantic salmon pre fed with a low-level TTA supplemented diet during natural outbreaks of viral diseases in sea [10,13], indicate important immune modulatory effects of TTA. Hence, TTA may be of interest as a feed additive in Atlantic salmon aquaculture.

To address immune modulatory functions of TTA in Atlantic salmon we conducted a "genome wide" microarray approach in a factorial designed experiment to identify transcriptional effects of TTA using the established, macrophage-like cell line ASK [14]. The cell line was chosen because macrophages pose a key role in the innate immunity, which is considered to be of primary importance to fight off pathogens since it can take weeks to mount an acquired immune response in poikilothermic fish [15]. Macrophages posses a remarkable plasticity that allows them to change their phenotype according to environmental signals. *In vitro *two major macrophage phenotypes have been characterized. Classically activated macrophages displaying a pro-inflammatory profile and alternative activated macrophages showing anti-inflammatory properties [16]. Macrophages recognize invading pathogens via pattern recognition receptors that bind conserved pathogen-associated molecular patterns (PAMPs). Activation of macrophages via PAMPs induces the classical activated phenotype. Because most viral pathogens produce dsRNA during one part of their replication [17], we decided to use the double stranded RNA analogue poly(I:C), a frequently used PAMP to simulate infection with a viral pathogen in our experiment.

A large proportion of the genes regulated by TTA was related to increased lipid metabolism and were also activated by TTA in poly(I:C) elicited ASK cells. The main effect seen for TTA in poly(I:C) elicited cells was to antagonize the induction of a subset of poly(I:C) dependent genes. Further we found increased expression of anti-inflammatory *IL10 *and *Krueppel-like factor 11 *(*KLF11*). Thus the results indicate that TTA polarizes the macrophage-like cells towards the alternative activated state.

## Results

We used the permanent Atlantic salmon macrophage-like cell line ASK in a factorial designed experiment to study transcriptional effects of TTA. An overview of the experimental design is given in Table 1. RNA from two biological replicates was used in the microarray analysis with the Atlantic salmon SIQ2.0 microarray [18] in a one-color setup, resulting in totally 16 arrays. One array did not pass the quality control analysis and was subsequently excluded from the final data set (Table 1, indicated by*). To identify differentially expressed (DE) probes, a linear model (Equation 2), containing the main effects was fitted to each probe set. Subsequently we extracted specific contrasts (Table 2), using the Bioconductor package "limma" [19].

**Table 1:** Experimental design

Pretreatment	Treatment	biol. repl.^a^	array^b^
Control	Control	4	2
	TTA	4	2
	TTA+poly(I:C)	4	2
	poly(I:C)	4	2

TTA	Control	4	2
	TTA	4	2
	TTA+poly(I:C)	4	2
	poly(I:C)	4	2*

^a ^Replicates, using different passages of the cells

^b ^Number of replicates used in the array experiment

* One of the arrays did not pass the quality control and was excluded from further analysis

**Table 2:** Specific contrasts

Contrast	*H*_0 _to test	Interpretation when *H*_0 _is rejected and the corresponding log_2_FC passes the cut-off
*preTTA vs. CTR *	*H*_0 _: *P_TTA _*= *P_contr _*	DE in: Samples pretreated with TTA *vs*. pretreatment Control
*TTA vs. CTR *	*H*_0 _: *T_TTA _*= *T_contr _*	DE in: Samples treated with TTA *vs*. Control treated
*TTAIC vs. CTR *	*H*_0 _: *T_TTA_*_+*polyI:C *_= *T_contr _*	DE in: Samples treated with TTA+poly(I:C) *vs*. Control treated
*IC vs. CTR *	*H*_0 _: *T_polyI_*_:*C *_= *T_contr _*	DE in: Samples treated with poly(I:C) *vs*. Control treated
*IC vs. TTAIC *	*H*_0 _: *T_TTA_*_+*polyI*:*C *_= *T_polyI_*_:*C *_	DE in: Samples treated with TTA+poly(I:C) *vs*. poly(I:C) treated

Contrasts, extracted for each probe after the full linear model (Equation 2) was fitted to the data.

Pretreatment with TTA (*preTTA vs. CTR*) significantly altered the expression of 121 probes (108 genes), while treatment with TTA (*TTA vs. CTR*) affected 49 probes (41 genes) (Table 3). Stimulating the cells with poly(I:C) showed the strongest effect with regard to number of DE probes and log_2_FC with 3636 DE probes (2785 genes) in the contrast *IC vs. CTR*. We observed significant alteration of 3487 probes (2697 genes) when treating the cells with TTA and poly(I:C) simultaneously (*TTAIC vs. CTR*). 92 probes (70 genes) were DE when comparing the concordant TTA and poly(I:C) treatment *vs*. poly(I:C) treatment (*IC vs. TTAIC*), indicating that TTA exerts effects on transcription in poly(I:C) challenged cells.

**Table 3:** Summary of significant probes identified by the different contrasts

Contrast	UP/DOWN	Probes	Probes **(lfc ***>***1)**	Probes **(lfc ***>***2)**
**Pretreatment**				
*preTTA vs. CTR*	UP	119	4	0
	DOWN	2	0	0

**Treatment**				
*TTA vs. CTR*	UP	27	6	0
	DOWN	22	1	0
*TTAIC vs. CTR*	UP	1043	476	250
	DOWN	2444	764	72
*IC vs. CTR*	UP	1491	594	227
	DOWN	2145	787	80
*IC vs. TTAIC*	UP	22	4	0
	DOWN	70	9	0

In this study we were primarily interested in transcriptional effects related to TTA. Thus, we will focus for the rest of the paper on the three TTA related contrasts: *preTTA vs. CTR*, *TTA vs. CTR *and *IC vs. TTAIC*. However, a comprehensive list of all DE probes for each contrast can be found in Additional file 1, Table S1.

**Figure 1:**
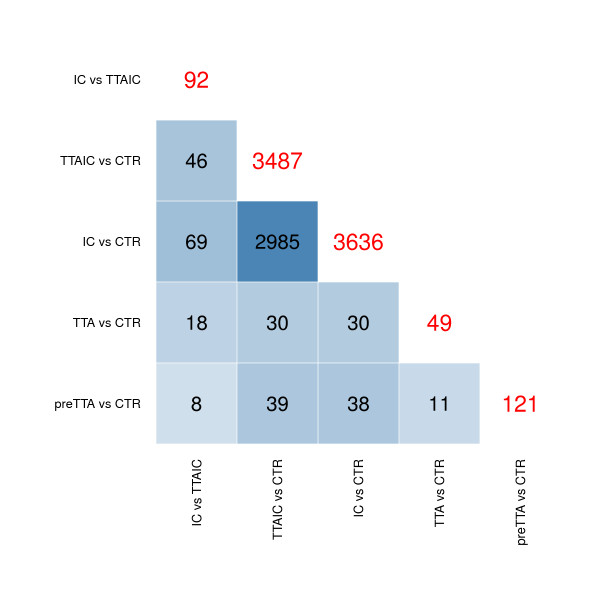
**Overlap matrix**. Overlap of the DE probes between the different contrasts. The numbers within cells display the number of common probes, while the red numbers in the diagonal display the total number of DE probes in that contrast.

The number of DE probes and their overlap between the different contrasts is shown in Figure 1.

### Transcriptional effects in IC vs. TTAIC

**Figure 2:**
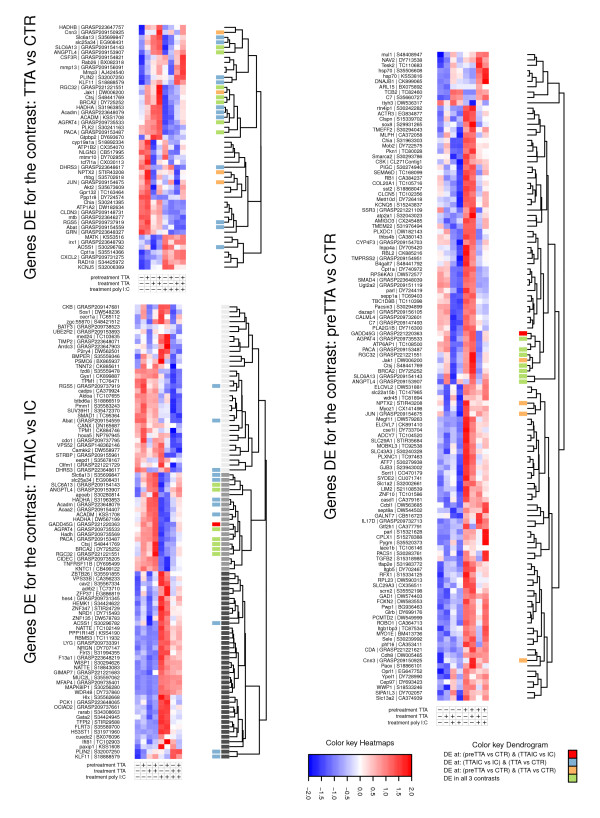
**Heatmaps**. Heatmaps displaying the relative expression of the probes significant in the three TTA related contrasts (*IC vs. TTAIC*, *TTA vs. CTR *and *preTTA vs. CTR*. The probes were clustered hierarchical using euclidean distance as indicated by the dendrogram on the side. The color code attached to the dendrogram indicates overlap for the corresponding gene between the 3 contrasts. In the dendrogram of the heatmap for contrast *IC vs. TTAIC*, the three major clusters are highlighted additionally (lightgrey - cluster.1; grey - cluster.2; darkgrey - cluster.3).

92 probes were DE when comparing ASK cells treated with poly(I:C) alone *vs*. cells that received concordant TTA and poly(I:C) treatment (Figure 1 and 2). Considering that poly(I:C) treatment alone significantly altered the expression of 3636 probes (Figure 1), the effects of TTA under poly(I:C) elicitation may be considered relatively small.

We observed that after hierarchical clustering of the DE probes for the *IC vs. TTAIC *contrast the genes could be separated into three major clusters (Figure 2). First, probes showing mostly a moderate down-regulation in response to poly(I:C), but a generally stronger down-regulation upon concordant TTA poly(I:C) treatment (cluster.1). Second, probes that were up-regulated by TTA, but down-regulated by poly(I:C) treatment (cluster.2). And third, probes that were less up-regulated when treated with TTA in combination with poly(I:C) (cluster.3). Especially for the second and the third cluster of probes we observed profound antagonistic effects for TTA in poly(I:C) elicited macrophages.

We found that cluster.2 contained mostly genes that were related to fat metabolism, and that most of them were shared with the *TTA vs CTR *contrast (see next section). Genes of particular interest were: *ANGPTL4*, as the highest ranking gene, *Acadm*, *AGPAT4*, *THIM*, *HADHA*, *PLIN2 *and *KLF11*. Angiopoetin-related protein 4 (ANGPTL4) has been reported, beside having a role in regulating angiogenesis, to act as a important stimulator of lipid metabolism [20]. Acetyl-CoA acyltransferase THIM and the mitochondrial trifunctional enzyme HADHA are enzymes of the mitochondrial *β*-oxidation. The gene *AGPAT4 *encodes an acyltransferase, participating in de-novo phospholipid synthesis. The gene remains yet to be fully characterized, however, other isoforms have been reported to be regulated by PPAR-*α *[21]. The protein Perilipin 2 (PLIN2) is a lipid droplet associated protein, recently suggested to stimulate lipolysis in macrophages [22]. Moreover, KLF11 an inhibitor of cellular PGE_2 _synthesis [23] and APOEB, the teleost homolog to apoE, was found to be strongly up-regulated. PGE_2 _is an important mediator of inflammation and has been previously reported to be down-regulated in response to TTA treatment [12,24]. ApoE secreted by macrophages participates in the regulation of cholesterol efflux, reducing the cholesterol-ester accumulation in macrophages attached to the arterial wall [25]. In addition, ApoE in macrophages has been reported to act as suppressor of pro-inflammatory signals and vice versa [26], and is reported to respond to PPAR-*γ *agonists in macrophages [27]. Hence, these results suggest that TTA induces lipid metabolism and also affects lipid mediators of inflammation in poly(I:C) elicited macrophages.

The genes in cluster.1 and cluster.3, down-regulated for the *IC vs. TTAIC *contrast, contained several genes with reported functions in immune modulation. The genes encoding Nattectin (NATTE) and Lysozyme G (LYG) both showed a strong increase in transcription in response to poly(I:C), which was attenuated when TTA was added. Nattectin has been identified as C-type lectin in fish [28]. C-type lectins function as pattern recognition receptors, recognizing pathogens and may subsequently opsonize them for phagocytosis. C-type lectins are induced following infection with bacterial [29] and viral [30] pathogens in fish, hence, suggesting host defense properties. Lysozyme is primarily associated with the defense against bacterial pathogens through hydrolyzing *β*1,4 glycoside bounds of peptidoglucans in the bacterial cell wall [31]. Expression of the anti-inflammatory marker *Pituitary adenylate cyclase-activating polypeptide *(*PACA*) [32], was strongly decreased in the *IC vs. CTR *contrast, but showed increased expression in the *IC vs. TTAIC *contrast. A similar pattern was observed for Os-teoprotegerin (*TNFRSF11B*). Osteoprotegerin is a member of the tumor necrosis receptor superfamily. Initially Osteoprotegrin was characterized for its ability to suppress osteoclast formation [33]. However Osteoprotegrin has also been reported to have a role in controlling inflammation. Exposure of mammalian endothelial cells to inflammatory cytokines has been reported to increase Osteoprotegrin expression, which in turn promotes leucocyte adhesion *in vitro *and *in vivo *[34].

### Transcriptional effects in TTA vs. CTR

49 probes were DE in response to treatment with TTA (Figure 1, Figure 2). From these 49 probes we found 27 to be up- and 22 probes to be down-regulated. The transcriptional changes in general were modest, with 6 probes showing an absolute log_2_FC ≥ 1 (Table 3). As shown in Figure 1 (and indicated in the dendrogram of Figure 2) the overlap to the previously described *IC vs. TTAIC *contrast was 37%, demonstrating that TTA affects a slightly different subsets of genes when applied alone to ASK cells.

Treatment with TTA generally increased the expression of genes involved in lipid metabolism. The gene showing the highest log_2_FC in this contrast was *Angiopoetin-related protein 4 *(*ANGPTL4*). Further we found a couple of genes encoding crucial enzymes for mitochondrial *β*-oxidation. We found *carnitine palmitoyltransferase 1A *(*Cpt1a*), the acyl-CoA dehydrogenase (*ACADM *) and mitochondrial trifunctional enzyme (*HADHB*) to be up-regulated. Also we observed increased expression of the gene encoding the lipid particle associated protein PLIN2 and short-chain dehydrogenase/reductase DHRS3. Thus, indicating increased lipid metabolism in ASK cells following TTA treatment.

We observed TTA regulation of a number of genes associated to anti-inflammatory effects. The second highest ranking gene up-regulated was the PGE_2 _inhibitor *KLF11 *[23]. Moreover we observed attenuated transcription of *Grannulins *(*GRN *)/*progrannulin *and the neuropeptide *pituitary adenylate cyclase-activating polypeptide *(*PACA*). Both genes are reported to exhibit anti-inflammatory effects. Macrophages isolated from mice deficient for the *Grannulin *gene showed higher transcription levels of pro-inflammatory cytokines [35], indicating an anti-inflammatory role for progrannulin. PACA has been identified to exert anti-inflammatory effects [32]. Increased expression of the matrix metalloproteinases *MMP3*, *MMP13 *and *chemokine ligand 2 *(*CXCL2*) was found in TTA treated ASK cells. Matrix metalloproteinases (MMPs) possess pleiotropic functions such as acting as matrix degrading enzymes, immunomodulary functions and have been suggested to be required for the migration of inflammatory cells [36]. CXCL2 has been implicated to act as chemotactic or activating factor for monocytes [37] and was further found to be highly induced by poly(I:C) treatment. Thus suggesting an increased potential for cell migration induced by TTA.

Further, *megakaryocyte-associated tyrosine kinase *(*MATK *) and the transcription factor *AP-1 *(*JUN *) proofed to be down-regulated. MATK is a nonreceptor tyrosine kinase that negatively regulates the activity of the Src family protein kinases - key regulators in signal transduction. A study showed higher levels of interferon-*γ *in MATK K.O mice challenged with antigens, thus suggesting a function for MATK in the immunological response [38]. JUN is an important transcription factor responding to cytokines and stress and has been reported to be up-regulated in response to viral challenges in Atlantic salmon [39,40]. Further we found a decreased expression of *NPTX2*, a member of the pentraxin superfamily that are known to act as pattern recognition receptors and can facilitate pathogen recognition and removal [15].

### Transcriptional effects in preTTA vs. CTR

**Figure 3:**
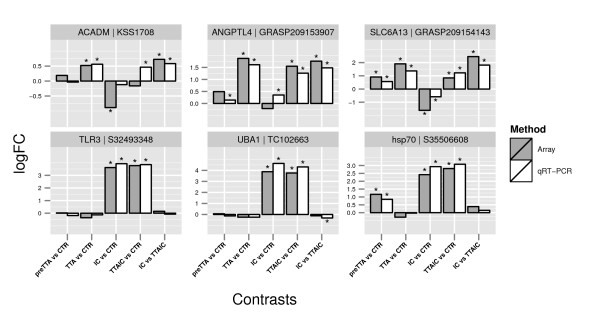
**qRT PCR verification**. Comparison of the log_2 _fold changes and the estimated significances between qRT-PCR (*n *= 4) and array (*n *= 2). Significances for the different contrasts were estimated by linear models for the array (as described in Methods) and 1-way ANOVA (Equation.1) followed by a Tukey test for qRT-PCR. Before calculating the fold changes the qRT-PCR expression values were normalized to the expression of the housekeeping gene Elongation factor 1*α *(EF1*α*). Contrasts found to be significant are marked with an asterisk. **ACDAM**: acyl-Coenzyme A dehydrogenase, medium-chain; **ANGPTL4**: Angiopoetin-like protein 4; **hsp70**: Heat shock protein 70; **SLC6A13**: Solute carrier family 6 member 13/sodium- and chloride-dependent GABA transporter 2; **TLR3**: Toll-like receptor 3; **UBA1**: ubiquitin-like modifier activating enzyme 1.

In the two experiments showing lower mortality in Atlantic salmon fed with TTA upon natural (viral) disease outbreak, the TTA feeding period ended before the onset of disease related mortality [10,13]. So we speculated that TTA might have long-term effects on the immunity in Atlantic salmon. To identify these effects we included the factor pretreatment in the experimental design. We found that pretreatment with TTA caused DE of 121 probes, which with the exception of two probes, all showed increased transcription (Table 3). The list of DE probes showed relatively little overlap with the two previously described TTA contrasts (7% and 9% to the contrasts *IC vs. TTAIC *and *TTA vs. CTR *respectively, Figure 2). Hence, suggesting that TTA pretreatment affects a different subset of genes. However, 38 of the probes in this contrast were also DE in the contrast *IC vs. CTR *(Figure 1). It should be noted that, similar to the effects observed in *IC vs. TTAIC*, the gene regulation in response to TTA pretreatment was mostly antagonistic to the effect found in *IC vs. CTR *(Figure 3).

Fatty acid metabolism and anti-inflammatory gene expression were less affected by pretreatment compared to treatment with TTA. The two fat metabolism related genes *ANGPTL4 *and *elongation of very long chain fatty acids protein 2 *(*ELOV2 *) were both up-regulated. Further we observed increased transcription of *Interleukin 17 D *(*IL17D*). The IL 17 family possesses an essential role in protecting the host from infection by pathogens [41]. The anti-inflammatory gene *PACA *and the pattern recognition receptor *NPTX2*, both described in the previous contrasts showed increased gene expression. It should be noted that the gene regulation of *NPTX2 *in *preTTA vs. CTR *was opposite to that found in *TTA vs CTR*. Two transmembrane proteins, Integrin *β *5 (ITB5) and thrombospondin-4 (thbs4b), both contributing to cell adhesion and migration showed increased transcription following pre-treatment with TTA. In addition we found increased expression of the transmembrane receptors *roundabout-1 *(*ROBO1*) and *plexin-C1 *(*PLXNC1*). Both receptors are considered to be repulsive receptors, contributing to the release of cell adhesion upon ligand activation [42,43]. These results may indicate that pretreatement with TTA supports increased cell migration in ASK cells, similar to the effects found in *TTA vs. CTR*.

Approximately 10% of the genes DE following TTA pre-treatment were transporters/ion channels. The genes included the GABA transporter (SLC6A13), Na^+^/dicarboxylate co-transporter (SLC13A2), SLC22A15b, SLC43A3, Cl^- ^transporter (CLCN5), v-type proton ATPase subunit s1 (ATP6AP1), ATP2A1, K-channel (KCNQ5), the neurotransmitter-gated ion channel GLRB and the Ca^2+ ^activated chloride channel TTYH3 as the only one showing down-regulation. CLCN5 and ATP6AP1 are responsible for the acidification of the endosomal lumen. Hence, the increased expression suggests a higher capacity for acidification of the endosome, which may promote phagocytosis [44].

### qRT-PCR

**Figure 4:**
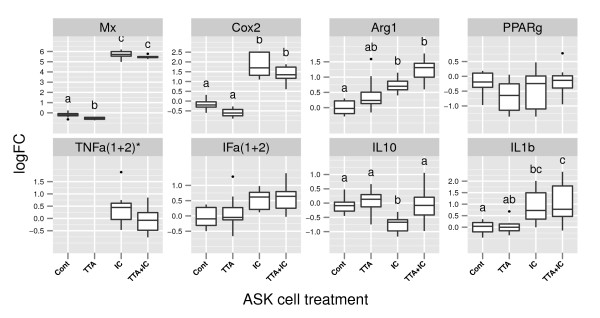
**qRT-PCR**. Box plots showing log_2 _gene expression relative to the mean expression of the samples that received control pretreatment and control treatment. Gene expression was normalized to the expression of the housekeeping gene EF1*α*. Significance was assessed using 1-way ANOVA (Equation.1). None of the tested genes was significant affected by TTA pretreatment. Different letters between the box plots indicate significant difference between the treatments, as determined by Tukey test (p-value ≤ 0.05, n = 4). **Mx**: myxovirus resistance 1; **Cox2**: Cyclooxygease 2; **Arg1**: Arginase 1; **PPARg**: peroxysome proliferator-activated receptor *γ *; **TNFa**: Tumor necrosis factor *α *(1+2); **IFa1+2**: Interferon *α *(1+2); **IL10**: Interleukin 10; **IL1b**: Interleukin 1β* Reliable gene expression could be only measured for samples that received poly IC treatment. Expression for TNF*α *is displayed relative to the samples that received control pretreatment and TTA + poly(I:C) treatment.

To validate the microarray results, six genes were randomly chosen from the list of DE genes for all contrasts. The genes were analyzed by qRT-PCR (Figure 4). In four cases we were able to detect significance by qRT-PCR, but not by array. However, in these cases the log_2_FC proofed to be lower than the cut-off of 0.5, that we used to identify significant genes in the array experiment. In one case we detected significance by array, but could not confirm this by qRT-PCR. Overall the results indicate that the parameters chosen to account for multiple hypothesis testing were appropriate. The results of the log_2_FCs for qRT-PCR matched those obtained by array. When comparing the log_2_FCs obtained for all genes and contrasts between qRT-PCR and array we found a highly significant correlation (pearson correlation of r = 0.96, p = 2.2e^-16 ^and an R^2 ^= 0.92).

To further study immune modulatory effects of TTA in ASK cells, we used qRT-PCR to measure the expression of selected genes previously reported to respond directly to TTA or other PPAR agonists (*Cox2*, *IL10*, *PPARγ*, *TNFα*, *Arg1*) and/or are important mediators of inflammation (*IL1β*, *IFα(1+2)*, *Mx*) (Figure 4). None of the analyzed genes was found to respond significantly to the effect of pretreatment with TTA. Several genes, however, responded significantly to the four different treatments.

For *Interleukin 10 *(*IL10*), which is generally considered to have anti-inflammatory properties, we found significant lower expression in the group that received poly(I:C), compared to all other treatment groups. The two pro-inflammatory cytokines TNF*α *and Interferon *α *showed no DE in response to the different treatments. It should be noted that the expression of *TNFα *could only be detected in poly(I:C) elicited samples and that concordant application of TTA and poly(I:C) reduced the mean expression by 50%. PPAR*γ *showed no altered expression in response to the different treatments. *Arginase 1 *was significantly higher expressed in all samples that received poly(I:C) treatment, additionally we observed that TTA also increased the *Arginase 1 *expression. The mean expression of the samples receiving TTA and poly(I:C) was 67% higher compared to the poly(I:C) samples. Even though not statistical significant, we observed a reduction in the expression of *COX2 *upon TTA treatment. Interleukin 1*β *is considered to be pro-inflammatory. We found a significant increase in transcription upon poly(I:C) elicitation, while concordant treatment with TTA and poly(I:C) elevated the mean expression (not significant). Mx is considered to be an antiviral protein [45], TTA treatment caused a significant reduction of *Mx *gene expression. Treatment with poly(I:C) increased the expression strongly.

## Discussion

In the present study we characterized the transcriptional responses to the PPAR activator TTA in the Atlantic salmon macrophage-like ASK cell line. Considering the strong effects documented for various PPAR agonists in different tissues like intestine or fat tissue (reviewed [46]) we found relatively few genes responding with altered transcription to TTA treatment. However, based on the results of the Microarray and qRT-PCR analysis TTA was found to stimulate the capacity for fat metabolism and have anti-inflammtory effects in the ASK cell line.

The innate immune response recognizes invading pathogens by pattern recognition receptors (PRR) through recognition of pathogen-associated molecular patterns. Toll-like receptors are a family of PRRs where each receptor displays specificity for it's lig-and [47]. Viral pathogens produce double stranded (ds) RNA during one part of their reproduction cycle. DsRNA is recognized by TLR3, triggering the production and release of pro-inflammatory cytokines [48]. The results from our microarray study showed that indeed a high number of genes were DE in poly(I:C) elicited macrophages. By comparing the group that received concordant treatment with TTA and poly(I:C) against the group that received poly(I:C) alone we identified a subset of genes showing antagonistic regulation. Several of these genes were sparsely characterized in the literature, especially in relation to immune function. However, the fact that we found those genes to be induced by poly(I:C) suggests a function in the Atlantic salmon immune response. Indicating that application of TTA to Atlantic salmon ASK cells has immune modulatory properties.

Expression of anti-inflammatory markers by macrophages is a hallmark for the alternative activated state. Anti-inflammatory effects of TTA have been mainly ascribed to increased expression of anti-inflammatory IL10. Indeed, we found a significant 98% increased transcription of *IL10*. Hence, indicating that TTA increases expression of *IL10 *in poly(I:C) activated macrophages, similar to the effects reported from human PBMCs stimulated with TTA [49,50].

Further we identified the PGE_2 _inhibitor *KLF11 *[23] as up-regulated in response to TTA. PGE_2 _is a mediator of inflammation, produced from PGH_2 _which is mainly produced by COX2. We observed that in poly(I:C) elicited ASK cells, TTA caused a mean reduction of 24% in *COX2 *gene expression. Arguably, these results indicate that TTA attenuates the capacity for production of pro-inflammatory PGE_2_, as it has been shown in primary macrophages from TTA fed fish reared at 5°C, but not for those kept at 12°C [12].

Arg1 competes with iNOS, an enzyme controlling the production of Nitric oxide for it's substrate. *Arg1 *is considered to be a key marker for alternative activated macrophages [51]. Recent studies in mice have shown that the expression of *Arg1 *in alternative activated macrophages is essential for suppressing inflammation [52]. Thus, our observation that TTA increased the expression of *Arg1 *by 67%, supports anti-inflammatory effects of TTA, and is also in accordance with a described regulation of *Arg1 *expression by PPAR-*γ *and *β*/*δ *[51]. Studies conducted on murine macrophages have shown that an up-regulation in fatty acid oxidation is crucial to direct them towards an alternative activated state [53]. In agreement with this we found that TTA induced the expression of genes that are related to lipid metabolism in ASK cells. Namely we identified increased expression of *Angiopoetin-like protein 4 *(*ANGPTL4*), *carnitine palmitoyltransferase 1 *(*Cpt1a*), *acyl-CoA dehydrogenase *(*Acdam*), *enoyl-Coenzyme A hydratase *(*HADH*) and the lipid droplet associated protein PLIN2. These effects of TTA are also consistent with reports of increased fatty acid oxidation upon TTA treatment [6,8] and also with effects associated to PPAR activation [5]. Taken together, the results provided by this study indicate that TTA directs the macrophage-like ASK cells towards an anti-inflammational, alternative activated state. Studies conducted in mice have reported that macrophages in the alternative activated state, via macrophage-parenchymal cell crosstalk, affect fat catabolism in liver and adipose tissues [54,55]. This effect was especially found to be important in animals under high-fat diet. Thus, we may speculate that TTA directed macrophage activation in Atlantic salmon fed high fat diets could contribute to an improved metabolic and inflammatory state.

Many studies have shown that the immune response is necessary in order to evict an invading pathogen, however, an excessive inflammatory response can cause unintended tissue damage, finally causing mortality [56]. Studies challenging Atlantic salmon with the viral pathogen ISAV (infectious salmon anemia virus) have shown that survival is not related to a strong innate inflammatory response, but rather to an attenuated innate and a stronger cell-mediated immune response [40]. Hence, it appears possible that the reduced mortality as reported from TTA fed Atlantic salmons during natural disease outbreaks [10,13] may partly result from anti-inflammatory effects of TTA.

TTA has been reported to act as an agonist for all PPAR subtypes *in vitro *[3,4], and it is assumed that most physiological effects of TTA are mediated via activation of PPARs. In our experiment we found neither significant transcriptional regulation of *PPAR-α*, *β*/*δ *(represented on the Array, data not shown) nor *PPAR-γ*, (Figure 4). However, the fact that a large proportion of the genes identified in our study have been reported to be regulated by PPARs and that the list of DE genes shows considerable overlap with the results from PPAR stimulated murine macrophages K.O for PPAR-*γ *or *β*/*δ *[57] support our assumption that the ASK polarization towards an alternative activated state may be induced via activation of PPAR-*γ *and *β*/*δ *by TTA.

## Conclusions

The results presented in this study suggests that TTA directs the macrophage-like cells towards an alternative activated state which is indicated by a stimulated lipid metabolism and attenuated inflammatory response. Further the pattern of TTA regulated genes indicate that the stimulation occurs via PPAR activation. The effects confirm a function of TTA in Atlantic salmon macrophages similar to those documented in mammals. These findings supports that TTA modulates the immune system of Atlantic salmon.

## Methods

### Cell Culture

The Atlantic salmon kidney cell line (ASK: ATCC: CRL-2747) [14] was cultured and propagated at 20°C according to standard cell culture procedures. Compared to mammalian cell lines, fish cell lines thrive slowly [58], so the ASK cells were studied for the respectively long period of 7 days. The cells were seeded out at day 1 in 6 well format (density 5 × 10^4 ^cells/ml). On day 2-4 the cells were pretreated with either TTA or control medium. Day 5-7: cells were treated with either Control, TTA, TTA+poly(I:C) or poly(I:C) medium.

Growth medium was L-15 (Invitrogen, MD, USA) supplemented with 10% FBS (Invitrogen), 40*μ*M *β*-Mercaptoethanol and 50*μ*g/ml Gentamycin. TTA medium was prepared by dissolving TTA (ThiaMed-ica, Norway) in 0.1 M NaOH at 80°C to a concentration of 100 mM, this stock-solution was diluted to a final concentration of 100*μ*M in growth-medium [59]. Control-medium was obtained by adding the same volume of 0.1 M NaOH as used in the TTA medium to the growth medium. Poly I:C was prepared by adding poly(I:C) (invitrogen) to a final concentration of (50 *μ*g/ml) to Control-medium. TTA+poly(I:C) medium was obtained by adding poly(I:C) to a final concentration of (50 *μ*g/ml) to the TTA-medium.

The cells were harvested on day 7 for RNA extraction. An overview of the experimental design is given in Table 1. The experiment was repeated four times with four different passages (passages 6-9) from the cells.

### RNA extraction

Total RNA was extracted using Qiazol (Qiagen, Halle, Germany) and purified from the cells using column purification (Qiagen) according to the manufacturer's instructions. Traces of genomic DNA in the samples were eliminated by on-column-DNase (Qiagen) digestion. RNA concentrations were measured for all samples using a NanoDrop 1000 Spectrophotometer. RNA quality for samples later used in the Microarray was determined using a Agilent 2100 Bioanalyzer (RNA 6000 NanoLabChip, Agilent, CA, USA). All samples had RIN values ranging from 9.6 to 10, indicating high quality of the RNA. Only individual RNA samples were used for subsequent qRT-PCR and microarray experiments.

### Quantitative RT-PCR

Single strand cDNA was reverse transcribed from 500 ng of total, DNase treated RNA using oligo dT primers and the Taq Man reverse transcription Kit (Applied Biosystems, Tx, USA). qRT-PCR was performed in 96-well optical plates on a Light-Cycler 480 (Roche, Switzerland). For the PCR reaction 2× SYBR green I master Mix (Roche), 0.41 nM of each primer and the cDNA template were mixed in a total reaction volume of 10*μ*l. Primer sequences are listed in Table 4. A three step PCR protocol with 45 cycles (15 s 95°C, 15 s 60°C, 15 s 72°C) was used. To verify specific amplification a melting curve analysis step was done at the end of the program. All samples were analyzed in duplicates and for each measured gene a standard curve was produced using a serial dilution from a pool of all cDNA samples.

**Table 4:** qRT-PCR primer sequences

**ID/Gene.Acc.Nr**.	Gene	Forward primer	Reverse primer
AF321836	EF1*α*	CACCACCGGCCATCTGATCTACAA	TCAGCAGCCTCCTTCTCGAACTTC
KSS1708	ACADM	GCCATCTCAGCCAACAGGAA	GACAAGACCAGACCGCCAGT
GRASP209147493	S6A13	CCGTATGGGGGATGATGCTAA	GGTAGTATGCTGACGACTGACACCT
S35506608	HSP70	AAGGCAAGATCAGCGAGGAG	TGCCTGATCTCCACAGCAAC
GRASP209153907	ANGL4	AGGAGATGCAGCAGGAGAGG	TTCTGAGCTTCCAGCATCCA
S32493348	TLR3	TCCAAGCCTGGTGACTCTCA	CAGGTGGAGGACTCGTTAGGG
TC102663	UBA1	GCACTGTTTCCCCTCTGACC	AGCAGTCTCGTGTTCCCACA
AF321836	TNF*α *(1+2)	GCTTGTCTCTTGTTGCCACCA	TGTGTGGGATGAGGATTTGGTT
AF088999	COX2	ACCTATGGGAACCGCAGGA	GTGAAGTGCTGGGCGAAGA
EG929369	ARG1	AGCCATGCGTATCAGCCAA	AAGGCGATCCACCTCAGTCA
AJ416951	PPAR*γ*	CATTGTCAGCCTGTCCAGAC	TTGCAGCCCTCACAGACATG
EF165028	IL10	ATGAGGCTAATGACGAGCTGGAGA	GGTGTAGAATGCCTTCGTCCAACA
AY216594+AY216595	IF*α *(1+2)	TGCAGTATGCAGAGCGTGTG	TCTCCTCCCATCTGGTCCAG
SSU66477	MX	TGATCGATAAAGTGACTGCATTCA	TGAGACGAACTCCGCTTTTCA
AY617117	IL1*β*	AGCAGGGTTCAGCAGTACAT	CTCCATAGCCTCACTCATCA

The expression levels were calculated using the standard curve method (Applied Biosystems User Bulletin 2). Expression levels were standardized to the expression of the housekeeping gene Elongation factor 1*α *(EF1*α*, [60]), and log_2_FCs were calculated relative to the average standardized sample that received control pretreatment and control treatment. Significance was tested by conducting a analysis of variance (ANOVA) test on the log_2_FCs for each gene using the model:(1)

Where *y_ijk _*is the log_2_FC of the *i^th ^*sample that received pretreatment *j *and treatment *k. P_j _*is the effect of pretreatment *j = *{*contr, TTA*}, *T_k _*is the effect of treatment *k = *{*contr, TTA, TTA+polyI:C, polyI:C*}, *μ *is a constant and *ε *is the error. A Tukey test was used subsequently to identify differences within significant effects.

### Microarray hybridization

The customized 4 × 44 K oligo (60-mer) Atlantic salmon microarrays SIQ2.0 (Agilent) [18] was used in the experiment. Unless otherwise stated all equipment and reagents were from Agilent. Amplification and labeling of 500 ng of total RNA and spike-ins for each sample was performed using the Quick Amp Labeling Kit (One-Color). After fragmentation of the Cy3 labeled cRNA, the samples were hybridized to the microarray at 65°C for 17 h. After hybridization the arrays were washed with Gene Expression wash Buffer 1 and 2 and scanned using a Agilent Microarray Scanner. All steps were conducted according to the Agilent protocol (One-Color Quick Amp Labeling, Version 5.7). Analysis of the mi-croarray images was done in Agilent Feature Extraction (Version 10.7.1.1) Software using the one-color (GE1_107_Sep09) protocoll. Array quality was assessed through Agilent control features and spike-in controls.

### Data analysis

One array did not pass the quality checks and was excluded from subsequent analysis. Normalization and analysis of the data was done in R/Bioconductor [61] using the Bioconductor package "limma" [19]. To generate the data-set, background corrected spot intensity signals (gProcessedSignal) were filtered according to the following criteria provided by Feature Extraction: gIsFound, gIsPosAndSignif, gIsWellAboveBG and ControlType (a description of the feature results can be found in the Feature Extraction Software Reference Guide). All Control spots were removed from the data set. The mean of the intensity signal for the duplicated features was calculated and the data was subsequently normalized by quantile normalization in order to adjust the scale of intensities across arrays [62]. The data was then log_2 _transformed and probe sets showing more than two missing values were discarded from the data set, classifying 12496 (59%) probe sets as present. Processed and raw microarray data is publicly available at NCBI's GEO repository (http://www.ncbi.nlm.nih.gov/geo/, Acc.Nr: **GSE25328**). A linear model (using zero intercept parameterization) was fitted to each probe set, where *y_ijkl _*is the log_2 _intensity of the *i^th ^*sample that received pretreatment *j *and treatment *k. P_j _*is the effect of pretreatment *j *= {*contr, TTA*}, *T_k _*is the effect of treatment *k *= {*contr, TTA, TTA+polyI:C, polyI:C*}, *B_l _*is the batch effect *l *= {*1,2*}, *μ *is a constant and *ε *is the error.(2)

Subsequent to fitting the model to each probe set, a number of specific contrasts of interest (listed in Table 2) were extracted for each probe set. For a graphical representation of the samples that were compared by the contrasts see Additional file 2, Figure S1. Improved variance estimates for the contrasts were computed using empirical Bayes moderated t-statistics from the "limma" package [63]. Before accounting for multiple hypothesis testing, probe sets without annotation were removed, reducing the data set to 10627 (50%) probe sets. Multiple hypothesis testing was accounted for by controlling the false discovery rate (FDR) [64] across all probes and contrasts simultaneously (using the method: global in the decideTests function from the 'limma" package). Probes were declared significant when the *q*-value was ≤ 0.15 and the log_2_FC ≥ 0.5.

Annotations for the probes were obtained using the function topBlast from the program Blast2GO [65]. Full length cDNA sequences of the probes have been compared in a BlastX search against the Swissprot protein database using an E-value cut-off of 10^-6 ^and the default program settings. All plots were generated using the R package "ggplot2" [66] and heatmaps were produced using "lattice" [67].

## Authors' contributions

FG designed and conducted the experiment, analysed the data and drafted the manuscript. HT participated in design of the study and writing the manuscript. Both authors read and approved the final manuscript.

## Supplementary Material

Additional file 1 **Table S1**. Table(.csv) of all probes. Column 1: Probe ID; columns 2-6: log_2_FCs of the corresponding gene for the 5 different contrasts *preTTA vs. CTR*, *TTA vs. CTR*, *TTAIC vs. CTR*, *IC vs. CTR *and *IC vs. TTAIC*. Column 7-9: Gene annotation, gene symbol and e-value. Column 10-14: significance of the corresponding gene for the corresponding contrast. Click here for file

Additional file 2 **Figure S1**. Contrasts: **A**: Figure of the experimental design (Table 1). Each of the eight nodes corresponds to a unique combination of the two experimental factors Pretraetment and Treatment. **B**: Figure displaying which combinations of Pretreatment and Treatment were used in the contrast *preTTA vs. CTR *(RED nodes vs. BLUE nodes). Figure for the remaining four contrasts are displayed; **C**: *TTA vs. CTR ***D**: *IC vs. CTR ***E**: *TTAIC vs. CTR ***F**: *IC vs. TTAIC*. Comparison is RED nodes vs. BLUE nodes, GREY nodes are not considered for the corresponding contrast. Click here for file
