# Supplementary figures and images for: Anti-inflammatory effects of tetradecylthioacetic acid (TTA) in macrophage-like cells from Atlantic salmon (Salmo salar L.)
Source: BMC Immunol. 2011 Jul 20;12:41. doi: 10.1186/1471-2172-12-41 (PMC3161001; doi:10.1186/1471-2172-12-41)

A

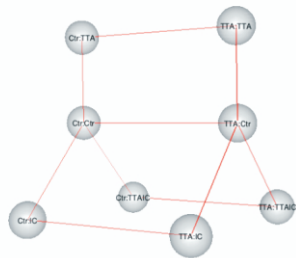

B

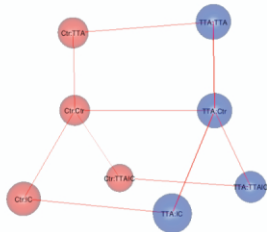

C

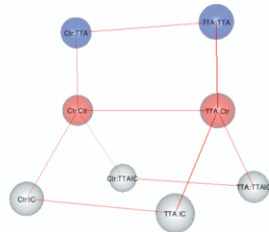

D

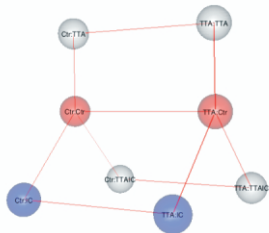

E

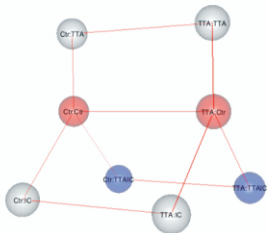

F

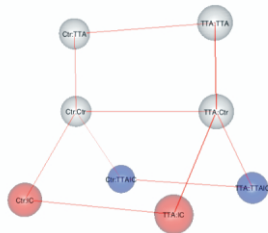

Supplement: Additional file 2 — Figure S1. Contrasts: A: Figure of the experimental design (Table 1). Each of the eight nodes corresponds to a unique combination of the two experimental factors Pretraetment and Treatment. B: Figure displaying which combinations of Pretreatment and Treatment were used in the contrast preTTA vs. CTR (RED nodes vs. BLUE nodes). Figure for the remaining four contrasts are displayed; C: TTA vs. CTR D: IC vs. CTR E: TTAIC vs. CTR F: IC vs. TTAIC. Comparison is RED nodes vs. BLUE nodes, GREY nodes are not considered for the corresponding contrast. [file 1471-2172-12-41-S2.PDF]
